# All-optical THz wave switching based on CH_3_NH_3_PbI_3_ perovskites

**DOI:** 10.1038/srep37912

**Published:** 2016-11-24

**Authors:** Kyu-Sup Lee, Rira Kang, Byungwoo Son, Dong-Yu Kim, Nan Ei Yu, Do-Kyeong Ko

**Affiliations:** 1 Department of Physics and Photon Science, Gwangju Institute of Science and Technology (GIST), Gwangju 61005, Republic of Korea; 2 Radiation Research Division for Industry and Environment, Korea Atomic Energy Research Institute, Jeongup, Jeollabuk-do 56212, Republic of Korea; 3 School of Materials Science and Engineering, Heeger Center for Advanced Materials, GIST, Gwangju 61005, Republic of Korea; 4 Advanced Photonics Research Institute, GIST, Gwangju 61005, Republic of Korea

## Abstract

Hybrid structures of silicon with organic–inorganic perovskites are proposed for optically controllable switching of terahertz (THz) waves over a broad spectral range from 0.2 to 2THz. A 532-nm external laser was utilized to generate photoexcited free carriers at the devices and consequentially to control the terahertz amplitude modulation, obtaining a depth of up to 68% at a laser irradiance of 1.5 W/cm^2^. In addition, we compared the performances from three types of perovskite devices fabricated via different solution processing methods and suggested a stable and highly efficient THz switch based on a one-step processing. By this we demonstrated the possibility of perovskites as THz wave switching devices in addition to photovoltaics.

Although over a couple of decades, tremendous progress in terahertz (THz) wave generators and detectors has been achieved, the realization of THz wave control devices is remained as a facing subject because studies of THz wave modulators and switches are still lagging behind. One approach for the development of THz wave switches has been based on the study of carrier-induced change in dielectric properties at simple structures of semiconductors^1^,^2^,^3^,^4^, and at hybrid structures of semiconductors with graphene^5^,^6^,^7^,^8^, metamaterials^9^,^10^,^11^,^12^, and organic–inorganic materials^13^,^14^,^15^. Zhang *et al*. have demonstrated optically controlled THz amplitude modulators with modulation depths (MD) of up to nearly 100% using a polymer and an organic film as deposited materials on a Si substrate with external laser pumping at a wavelength of 400 nm^16^,^17^,^18^.

Recent discoveries of photovoltaics in methylammonium lead halide perovskites (MAPbX_3_, where X = I, Cl, and Br) have significantly contributed to next-generation solar cells. Owing to their long electron–hole diffusion length (~100 nm for CH_3_HN_3_PbI_3_ and >1,000 nm for CH_3_NH_3_PbI_3−x_Cl_x_)^19^, low cost, and ease of fabrication with solution processing, perovskite-based solar cells have exhibited power conversion efficiencies of up to 20.5% experimentally^20^ and 31% theoretically^21^.

Moreover, there is additional scope for implementation of perovskite technology at THz frequencies, being of particular significance for THz switching. One recent report has shown an optically controlled THz amplitude modulator based on the hybrid structure of Si with a perovskite film fabricated via two-step solution processing^22^. However, the instability of perovskites under ambient conditions has remained problematic in practical applications^23^. Kang *et al*. have recently reported a comparative investigation of CH_3_NH_3_PbI_3_ perovskites according to their fabrication methods and identified that more suitable devices for practical uses are based on one-step solution processing owing to the narrow distribution of the device efficiency and the stability under ambient conditions^24^,^25^. In the present report, we conducted a quantitative study of perovskite-based THz switches. Hybrid structures of Si with perovskites (perovskite/Si) fabricated through three different processing methods were compared in terms of the MD, carrier density, conductivity, and mobility. Finally, we demonstrated a stable and highly efficient perovskite THz switch can be fabricated by a one-step processing method.

## Results and Discussion

**Figure 1:**
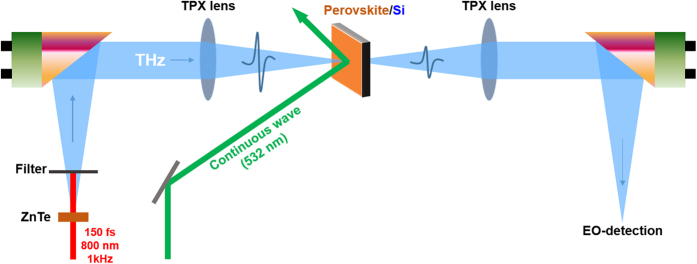
Schematic diagram of optically controllable THz modulation of perovskite/Si. The THz pulse was generated using a 1-mm-thick ZnTe crystal and was guided through two off-axis parabolic mirrors (each with a focal length of 100 mm) and two polymethylpentene (TPX) lenses (each with a focal length of 50 mm). The 532-nm cw laser illuminated the surface at an obilique angle of 45° with a spot size ~2 mm larger than a 1-mm THz spot.

In the present experiment, we utilized a typical THz time-domain spectroscopy (THz-TDS) system with a spectral range from 0.2 to 2 THz based on a regenerative amplified femtosecond pulse laser system (Hurricane, Spectra-Physics). To modulate the THz wave transmission, an external continuous wave (cw) laser with a wavelength of 532 nm was used to illuminate a THz switching device at an oblique angle of 45° with a larger beam size than a THz spot, as shown in Fig. 1. We took note that the measurement of the THz modulation with hybrid perovskite/Si devices at the wavelength of 532 nm has never been reported even with the fact that green light is the most abundant spectral component in sunlight. So, in contrast to previous research on perovskite-based modulators with weak THz pulse and 400-nm optical pump^22^, we chose 532 nm of wavelength as optical pump and modulated intense THz pulse, which will be the realistic tool to characterize perovskite materials as solar devices.

**Figure 2:**
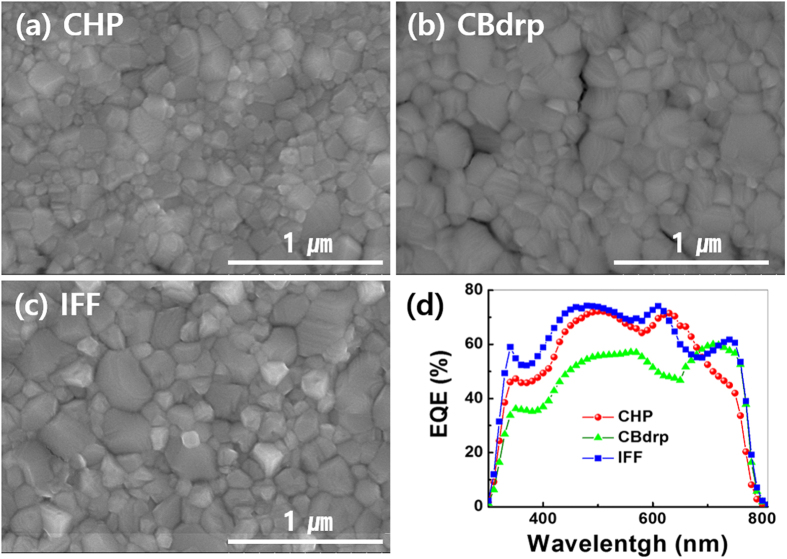
Features and electical properties of perovskite devices. Scanning microscope images of perovskite surfaces based on (**a**) CHP, (**b**) CBdrp, and (**c**) IFF fabrication methods, and (**d**) the corresponding external quantum efficiency.

Three types of CH_3_NH_3_PbI_3_ perovskites for THz wave switching were fabricated based on different representative solution-processing methods. The three processing methods were (1) CHP: one-step processing method with an additive, N-Cyclohexyl-2-pyrrolidone to the precursor solution, (2) CBdrp: one-step processing method by quickly dropping chlorobenzene on the spinning wet MAPbI_3_ films, and (3) IFF: two-step processing method with spin-coating by two precursor solutions, as explained in detail at ref. 25. Scanning electron microscope images of the perovskite surfaces were obtained with different morphologies (Fig. 2(a–c)), giving different modulation properties, which will be discussed later in this report.

Furthermore, external quantum efficiencies (EQEs) for three types of perovskites were obtained over a range from 300 to 800 nm as shown in Fig. 2(d). By the fact that the THz modulation is mainly affected by the number of generated free carriers^16^ equivalent to an EQE of a device, we estimated the relative MDs among three perovskite/Si devices with EQEs at a given 532-nm pump. The CHP- and IFF-based perovskites which show comparable EQEs of approximately 70% are expected to exhibit higher THz modulation than CBdrp-based perovskite with the EQE of 55%. Meanwhile, both CHP and CBdrp, one-step processing, are more stable under ambient conditions than IFF as mentioned above^25^, which therefore anticipates that CHP-based perovskite/Si device is the best candidate for THz switching at a 532-nm pumping owing to its good stability and high MD.

Several types of hybrid Si structures have exhibited higher and faster THz wave modulation compared to bare Si at low laser irradiance^14^,^17^,^18^, because energy band bending, which causes the drift of excited photocarriers towards the interface, occurs at the interface between Si and deposited materials. The conductivity at each region (Si, deposited materials, and the interface) naturally varies so that the photocarriers accumulate at the interface that gives a higher carrier density and an increase in THz wave absorption via electron–hole scattering, electron–phonon scattering, and electron–impurity scattering, which finally enhances the THz amplitude modulation than from both bare Si and the deposited material itself^16^. In this experiment, we used three types of CH_3_NH_3_PbI_3_ perovskites deposited onto a 500-μm-thick, high-resistivity (>1000 Ω cm), and undoped Si wafer. The conduction band edges from the vacuum state were −4.07, −4.08, −3.88, and −3.1 eV at CHP-, CBdrp-, and IFF-based perovskites and Si, respectively^25^. This mismatch of the band alignment between Si and each perovskite creates band-bending structures at the interface and causes the drift of the excited photocarriers^14^. The photocarrier mobility of perovskite (on the order of 10)^26^ is a factor of ~100 less than that of Si (on the order of 10^3^)^27^, causing an accumulation of photocarriers at the interface and, subsequently, an increase in the THz modulation. In addition, this photo-doping effect by the external laser pump is enhanced at higher irradiance, causing the corresponding higher THz modulation.

**Figure 3:**
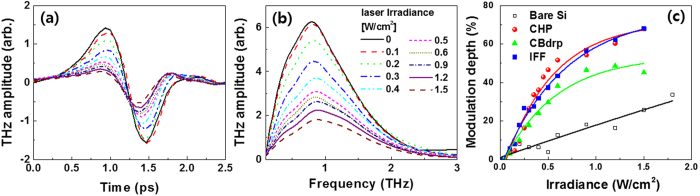
Optically controlled THz modulation results. The modulated (**a**) waveform and (**b**) spectrum of the CHP device and (**c**) the modulation depth at each device under various laser irradiances.

Figure 3(a) and (b) show the amplitude modulation of the waveform and spectrum in THz transmission under various laser irradiances for CHP-based perovskite/Si. THz transmission decreased as the laser irradiance increased. CBdrp- and IFF-based perovskite/Si and bare Si followed the CHP-perovskite/Si in the THz modulation with different levels. For quantitative analysis, we introduced the MD defined as the change in the integrated THz transmission spectrum as


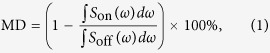


where *S*_*on*_*(ω)* and *S*_off_(ω) are the THz transmission spectra with and without the optical laser pumping, respectively. As expected, THz modulation for perovskite/Si devices was further enhanced comparing with bare Si, as shown in Fig. 3(c). The MDs for the three perovskite/Si devices were increased linearly at low irradiation and then saturated as the irradiation was increased (~0.5 W/cm^2^), whereas, for bare Si, the low linear growth of MD was observed within the experimental range (up to 2 W/cm^2^). We obtained an MD of up to 68% for both CHP- and IFF-based perovskite/Si devices at a laser irradiance of 1.5 W/cm^2^, although we used a 532 nm as an optical pump source relatively far from the peak absorption band near 400 nm in Si. The absorption coefficient of 7850 cm^−1^ in Si at 532 nm is a factor of ~4 smaller than the coefficient of 25500 cm^−1^ at 450 nm^28^, which indicates that the number of excited carriers involved in the THz modulation at 532 nm was highly decreased when compared to at 450 nm. Nevertheless, we observed the clear enhancement of THz modulation at perovskite/Si devices.

**Figure 4:**
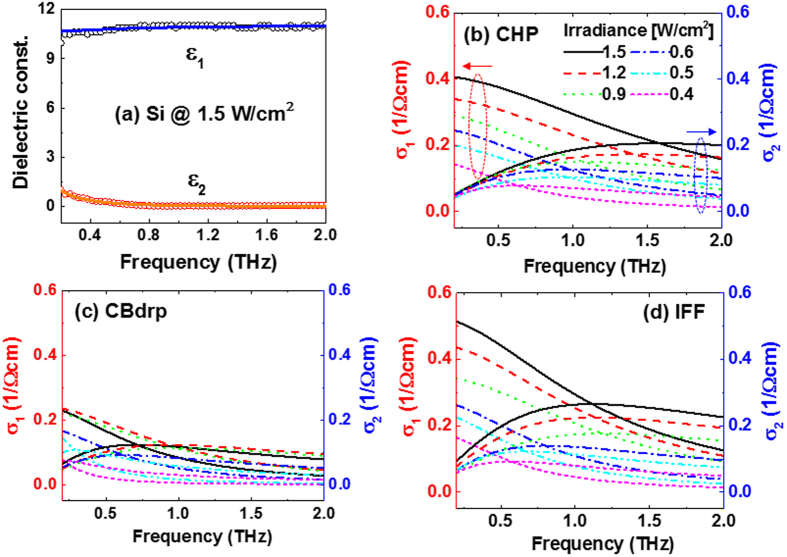
Freqeuncy-dependent complex constants extracted by THz-TDS based on the Gabor WT and the Drude model. (**a**) Complex dielectric constants of bare Si and the corresponding complex conductivities of (**b**) CHP, (**c**) CBdrp, and (**d**) IFF devices under various irradiances.

To investigate the modulation mechanism in detail, we deduced the optical constants of the perovskite/Si devices via THz-TDS under various laser irradiances. The complex dielectric constant was obtained by comparing the THz transmission at the perovskite/Si devices to bare Si. We extracted the refractive index and the absorption coefficient from the time-frequency spectrogram based on the Gabor wavelet transform (Gabor WT)^29^ to calculate the complex dielectric constant, *ε*_*complex* = _*ε*_*1*_* *+* iε*_*2*_, as shown in Fig. 4(a). The real and imaginary parts of dielectric constant were 10.86 and 0.10 at 0.75 THz, respectively, which are higher than those of 9.9 and 0.01 reported by Li *et al*.^30^. This can be explained by the fact that the photo-doping on Si by external laser pump in our experiment changed the dielectric property^31^.

Complex photoconductivity was extracted based on a simple Drude model as


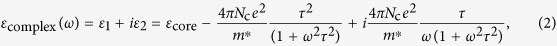


with


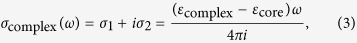


where *ε*_*core*_, equal to *ε*_*complex*_(∞), is the core dielectric constant, *N*_c_ is the photocarrier density, *e* is the electron charge, *m*^∗^ is the effective carrier mass, τ = 1/γ is the averaging collision time, and γ is the damping rate. The corresponding frequency-dependent photoconductivities for three perovskite/Si devices under various laser irradiances are shown in Fig. 4(b–d), being consistent with the previous reports on the conductivities of doped Si^31^,^32^.

**Figure 5:**
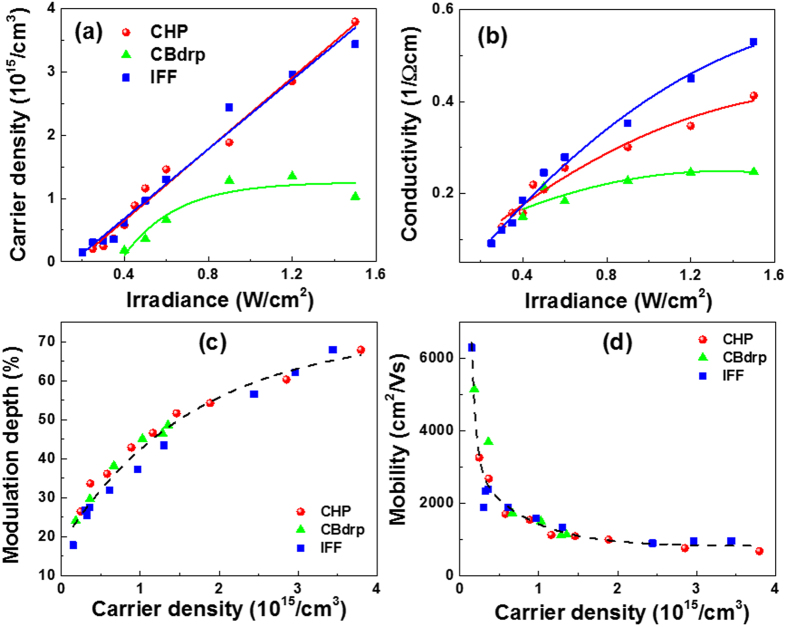
Frequency-independent electrical constants of perovsktie/Si devices. (**a**) Carrier density and (**b**) dc conductivity under various irradiances. (**c**) MD and (**d**) mobility according to the carrier density.

The electric constants were deduced from the Drude fit. By the photo-doping effect, the both carrier density (*N*_c_) and dc conductivity (σ_dc_) increased as the irradiance increased, as shown in Fig. 5(a) and (b), respectively. As we can expect from the EQEs in Fig. 2(d), CHP- and IFF-based perovskite/Si devices showed almost the same carrier density which is higher than that of CBdrp. Figure 5(b) shows dc conductivities for three perovskite/Si have different behavior to the irradiance, which is because the difference in morphology occurred during the fabrication, where crystal orientation, growth, and corresponding electronic properties differ^25^. This result implicates that the IFF-based perovskite/Si device with the highest σ_dc_ gives the highest enhancement in MD under an external electric bias^18^. In addition, we obtained the increase in MD with higher carrier density accordingly as expected (Fig. 5(c)). Figure 5(d) shows the change of mobility as a function of carrier density. At low carrier density regime (<10^15^/cm^3^), the photocarrier mobility showed a steep decline and, at high density regime, it asymptotically approached to the value of <1000 cm^2^/Vs, which was consistent with the mobility for Si given by Exter *et al*.^27^ and Nashima *et al*.^33^.

## Conclusion

We investigated the optically controllable THz switching properties for hybrid structures of Si with three organic–inorganic perovskites fabricated through the different solution-processing methods: CHP, CBdrp, and IFF. As we expected on high MDs of CHP-and IFF-based perovskite/Si devices from the EQE values, a maximum 68% of amplitude modulation was obtained for the both devices at an external 532-nm laser irradiance of 1.5 W/cm^2^, where the extracted maximum photocarrier density was 3.8 × 10^15^ cm^−3^ via THz-TDS and the Gabor WT. From this comparative study, the CHP-based perovskite/Si device fabricated with one-step processing showed good performance in its modulation efficiency and stability, which may assure that this device can be a good candidate for a practical THz switch.

## Methods

### Terahertz time-domain spectroscopy

The THz-TDS system utilized a regenerated amplified Ti:sapphire laser system (Hurricane, Spectra-Physics) with a time duration of 190 fs, a wavelength of 800 nm, and a repetition rate of 1 kHz. A typical THz-TDS setup was utilized with two ZnTe crystals for a THz source and electro-optic detection, providing a spectral range of 0.2–2 THz. Through time-frequency spectrograms based on the Gabor WT, we obtained the temporal delay on each frequency and the relative spectrum ratio by comparing the reference (from bare Si without optical laser pumping) and sample signal (from perovskite/Si with the pumping). The Gabor WT analysis increase an accuracy in calculation than from the Fourier transformation (FT), because the temporal delay in each spectral component was directly extracted to obtain a refractive index at the corresponding frequency instead of using the relative spectral phase shift that are typically used in FT which is sensitive to experimental conditions and gives an experimental error.

### Sample fabrication

Hybrid structures of Si with CH_3_NH_3_PbI_3_ perovskite were fabricated via three different solution-processing methods. Si substrates were rinsed with acetone, DI water and isopropyl alcohol, followed by UV-O3 treated for 20 min, and then CH_3_NH_3_PbI_3_ films were fabricated according to the procedure in the previous report^25^.

## Additional Information

**How to cite this article**: Lee, K.-S. *et al*. All-optical THz wave switching based on CH_3_NH_3_PbI_3_ perovskites. *Sci. Rep*. **6**, 37912; doi: 10.1038/srep37912 (2016).

**Publisher's note:** Springer Nature remains neutral with regard to jurisdictional claims in published maps and institutional affiliations.
